# Semantic and emotional content of imagined representations in human occipitotemporal cortex

**DOI:** 10.1038/srep20232

**Published:** 2016-02-03

**Authors:** Daniel J Mitchell, Rhodri Cusack

**Affiliations:** 1 MRC Cognition and Brain Sciences Unit, 15 Chaucer Road, Cambridge CB2 7EF, UK; 2 Brain and Mind Institute, University of Western Ontario, London, Ontario N6A 5B7, Canada

## Abstract

Mental imagery is a critical cognitive function, clinically important, but poorly understood. When visual objects are perceived, many of their sensory, semantic and emotional properties are represented in occipitotemporal cortex. Visual imagery has been found to activate some of the same brain regions, but it was not known what properties are re-created in these regions during imagery. We therefore examined the representation during imagery for two stimuli in depth, by comparing the pattern of fMRI response to the patterns evoked by the perception of 200 diverse objects chosen to de-correlate their properties. Real-time, adaptive stimulus selection allowed efficient sampling of this broad stimulus space. Our experiments show that occipitotemporal cortex, which encoded sensory, semantic and emotional properties during perception, can robustly represent semantic and emotional properties during imagery, but that these representations depend on the object being imagined and on individual differences in style and reported vividness of imagery.

The importance of mental imagery is clear from the range of cognitive functions it supports, including short-term memory storage^1^,^2^, long-term memory retrieval^3^, creative insight^4^, construction of attentional search templates^5^,^6^,^7^, simulation of future events^8^, perceptual biasing^9^,^10^, and driving willed action^11^. Mental imagery is disturbed in many clinical conditions such as neglect^12^,^13^,^14^, tunnel vision^15^, schizophrenia^16^,^17^ and William’s Syndrome^18^, and can be debilitating when uncontrolled, in post-traumatic stress disorder and phobia^19^,^20^. Recently, mental imagery has seen increasing use as a therapeutic technique to treat emotional disorders^19^,^20^, and as a clinical tool to assess conscious awareness when behavioural responses are unavailable^21^.

Despite this fundamental role in cognition, and increasing clinical importance, the information content that is represented during mental imagery remains elusive. From a neural perspective, progress in identifying the brain networks that support imagery has not been accompanied by an understanding of the neural content of a mental image. Recent neuroimaging has focused on visual imagery, finding similarities with perception, both in large-scale mean activity^22^,^23^ and fine-scale activity patterns^24^,^25^,^26^,^27^. These similarities are sometimes found in primary visual cortex^28^, but are more consistently observed in higher-level ventral visual regions. For example, in occipitotemporal cortex, classifiers trained on activity patterns evoked by perceived items were able to successfully classify imagined items, and vice-versa. This demonstrates that occipitotemporal cortex represents some common information across perception and imagery, however the nature of the shared information remains unclear. During perception, this region encodes a range of both simple sensory and higher-level object properties^29^,^30^,^31^,^32^. Existing studies of imagery have presented few objects (or object categories), and compared imagery of each object with perception of the same object. From these experiments, it is impossible to determine which object features drove object-specific responses during imagery, or contributed to the overlap with perception.

The neural instantiations of perception and imagery are not expected to be identical^33^, despite having some shared aspects as shown by the above studies. Perception and imagery dissociate clinically^13^,^34^,^35^,^36^, and they evoke responses that differ in their spatial distribution of deactivation^37^ and in their object-selectivity^27^. Therefore, an important open question is: How does the brain’s representation of imagined objects relate to the representational space of perceived stimuli? Which features are coded similarly, and which are abstracted?

To de-confound the many possible sensory, semantic and emotional features that might be represented, it is necessary to use a large set of objects. Thus, we compared the representation of single objects during imagery or perception with the representation of 200 other objects during perception. This allowed us to investigate which features were represented in a common way in occipitotemporal cortex during visual imagery and perception. Feature tuning was assessed using pair-wise, representational similarity analysis of multi-voxel fMRI activity patterns^38^ evoked by the 200 images. Using a rich stimulus set increases the number and sampling resolution of assessable feature dimensions, however, with limited scanning time, comparing every stimulus to every other stimulus becomes increasingly impractical. With imagery this is especially problematic, both because generating a mental image is slower than perception^39^, and because it is prohibitively challenging to memorise many stimuli sufficiently for subsequent vivid imagery. Therefore, rather than characterising the entire space of imagery representations, power was focused in each experiment on characterising the neural representation of a predefined “referent” image. To this end, pair-wise pattern similarity analysis was combined with real-time, adaptive stimulus selection^29^, to efficiently search the stimulus space: An iterative process resampled subsets of stimuli whose evoked activation patterns were most similar to that of the referent (Fig. 1, top). These stimuli were presented visually, so only the referent needed to be memorised and imagined, while other stimuli could be presented more rapidly. In this way, online neuroimaging was used to select a subset of stimuli that reflected the “neural neighbourhood” (NN) of the referent image. Then, by quantifying these stimuli along various sensory, semantic, and emotional dimensions, common properties of the final subset were examined in subsequent offline analyses (Fig. 1, bottom), to reveal features that they shared more than would be expected by chance.

## Results

### Occipitotemporal cortex encodes multiple semantic and emotional properties of perceived and imagined objects

The occipitotemporal region of interest was significantly active during both perception and imagery, as shown using an initial whole-brain univariate analysis (Fig. 2). However, subsequent multivoxel pattern analyses revealed important differences in the particular object features that were represented in this region during perception, and those that were represented during imagery.

To illustrate the representational space of the 200 stimuli within this region of interest, we used multidimensional scaling to represent their activations patterns, yielding a space in which objects that are closer have a more similar neural representation. Furthermore, to assess whether sufficient power was obtained within a single iteration of the real-time procedure, we calculated this from the first run alone (Fig. 3). Data from individual participants were optimally combined using DISTATIS^40^, and 95% confidence ellipses indicate variability. (The first eigenvalue of the cross-participant similarity matrix (λ_1_ = 64.2) explained 76% of the variance, indicating moderate consistency of representational spaces across participants^41^). It is clear that there is detailed information in the local activation patterns, even after just a single presentation of each item. Each stimulus is colour coded according to a recently proposed tripartite organization into animate items and large and small inanimate objects^42^. Strong pattern separation between animate and inanimate items is clear, as expected for this brain region, and separation between small and large objects is also observed along the vertical dimension. A robust degree of consistency is apparent; both across both participants (confidence ellipses) and conditions (compare the lower panels). (Despite the obvious feature information contained within the first two principal components, these components explained a mere 2.45% of the variance in the data (eigenvalues λ_1_ = 0.20, λ_2_ = 0.11). The remaining variance will reflect a mixture of other object features encoded in higher dimensions, and measurement noise, which will be substantial in the response at the level of single images given the large number of stimuli (200) but low number of repetitions.)

Having shown that the neural patterns contained representation of two exemplar features, and that the representational space was reproducible across subjects and conditions, we next confirmed that neural patterns were consistent across repetitions of an object within a subject. Standard MVPA classification (using a linear support vector machine with leave-one-run-out cross-validation) was used to decode the identity of stimulus pairs that were repeated in four or more runs. Mean classification accuracy (averaged across cross-validation splits and every possible stimulus pair) was compared to chance using t-tests across participants and confirmed to be significant for both conditions of both experiments (Knife referent, perceived: t(20)=3.20, p < 0.01; knife referent, imagined: t(21) = 3.15, p < 0.01; dolphin referent, perceived: t(20) = 2.95, p < 0.01; dolphin referent, imagined: t(20) = 2.24, p < 0.05). This shows the neural patterns contained information that allowed pairs of objects to be discriminated.

Given these quality assurance analyses, we moved on to address the core goal of this manuscript, to measure and compare which features of the referent items were neurally represented during perception and imagery. For the two experiments, Table 1 lists those images that evoked the most similar neural patterns, on average, to each perceived or imagined referent. If a particular feature of the referent (e.g. its blue colour) was strongly neurally represented, then many other images sharing that feature (i.e. other blue objects) would be members of the neural neighbourhood. We started by examining the broader picture, of whether sensory, semantic or emotional feature sets were represented. The results are shown in the left-hand bars of each panel of Fig. 4, labelled “Multivariate classifier sensitivity”. For each *perceived* referent (upper half of figure), a multivariate classifier predicted individual images in the neural neighbourhood (after training on the other images i.e. leave one out cross-validation), when provided with their sensory features (red bars; knives, p < 0.05; dolphin, p < 0.05), semantic features (blue bars; knives, p < 0.005; dolphin, p < 0.005) or emotional features (purple bars; knives, p < 0.05; dolphin, p < 0.005). These results show that there was rich encoding, of the sensory, semantic and emotional features of the referents during perception. For the *imagined* knife referent (experiment 1; lower left quadrant) semantic and emotional feature-sets were strongly encoded (semantic p < 0.005, emotional p < 0.005). The sensory feature set no longer predicted NN membership, although the feature set-by-condition interaction was not significant (p > 0.05). These results show that there is rich overlap in the neural representations that underlie perception and imagery, with strong evidence for common encoding of semantic and emotional features. In experiment 2 (dolphin referent), although classification during the perception condition was stronger than in experiment 1, classification during imagery (lower right quadrant) no longer reached significance for any of the three feature-sets. A significant feature-set-by-condition interaction (p < 0.01), showed that this was not due to a general lack of power, but that the dolphin was represented in a different way across perception and imagery. Some support for a difference in processing between the animate and inanimate referents was also apparent during perception: Consistent with previous findings^29^, classification of the perceived referent based on semantic features was especially strong for the animate dolphin referent, whereas sensory and semantic features had comparable predictive power for the inanimate knives referent (feature-set-by-referent interaction: p = 0.09).

For feature sets where significant classification was observed, we then drilled down, and identified which individual features were most strongly represented. The degree to which individual features predicted NN membership is shown in the centre column of each panel, labelled “univariate classifier sensitivity”. A few sensory features, and most of the semantic features, were represented during perception (upper half of figure). Furthermore, during imagery of knives (lower left quadrant), several semantic features were found to be strongly encoded (animacy p < 0.01, knife association p < 0.05, dolphin association p < 0.01, and implied motion p < 0.01). Sensitivity to real-world size and to object number were not preserved during knife imagery. Within the emotional features, predictive power of an image’s valence and arousal differed across perceived referents (valence significant for knife; arousal significant for dolphin), and just failed to reach significance during imagery for the knife referent.

This classifier analysis revealed which features significantly predicted NN membership (e.g., emotional valence), but not whether images with positive or negative values of each feature were being selected for membership. To investigate this, the rightmost bar charts in each panel (labelled “Mean feature value in NN”) plot the mean value of each feature, across the ten images comprising the NN. In general, the direction of significant tuning for a given feature was consistent with whether the referent image had a positive or negative score for this feature. For example, images with strong implied motion were consistently selected as having similar activation patterns to the dolphin, which was also rated highly on this feature, whereas objects with little implied motion were consistently selected as having similar activation patterns to the knives. Typically, features from which NN membership could be classified were present in the NN more or less than would be expected by chance. In particular, this was true of all four semantic features that allowed significant classification of NN membership during knife imagery. Also consistent with the classification measure, the two remaining semantic features (real-world size and object number) showed non-zero tuning during perception of knives but not during imagery of knives. This drop in tuning was significant for real-world size (t(20) = 2.50, p < 0.05) but not for object number (t(20) = 1.09, p > 0.1).

Occasionally, a feature that significantly predicted NN membership was not significantly expressed in the NN, which could reflect consistent selection of a stimulus value close to the average across the stimulus set, or selection of more extreme values but in different directions across participants. Conversely, during knife imagery, the NN contained images with significantly negative valence, despite valence not significantly predicting NN membership in the classifier analysis. This could reflect insufficient power of the classifier, or suggest that images with negative valence were clustered not only within the NN, but also elsewhere along the distribution of neural pattern similarities.

Finally, feature tuning of the neural representation during mental imagery was compared across the two referents. Again, coarsely grouped feature sets were considered first, and a significant interaction (p < 0.01) was found between feature set (sensory vs. semantic/emotional) and imagined object (knives vs. dolphin) in terms of ability to predict NN membership. To explore these differences in more detail, object-specific coding of individual features is plotted in Fig. 5, as their differential expression within the NN. For participants imagining the dolphin, images in the NN were significantly more blue, rated as less semantically associated with knives, more associated with dolphins, and arousing more positive emotions, compared to NN items for the participants who were imagining knives (all p < 0.05; Fig. 5, middle). These effects did not survive correction for multiple comparisons, but were all in the direction expected *a priori* based on the feature properties of the referents. For semantic features, these differences correspond to image properties that were also represented during perception (Fig. 5, left), consistent with the participants’ goal to recreate the perceptual experience during imagery. A significant interaction was found, however, between referent (dolphin vs knives) and condition (perceived vs imagined) for coding of animacy (p < 0.05; Fig. 5, right), which was positive during dolphin perception, negative during dolphin imagery, and negative during both knife conditions.

The broad stimulus set suits both theory-driven and exploratory analyses. Features considered above were chosen *a priori*, bounding the space of possible inferences. However, this brain region may be sensitive to properties not considered in advance. During dolphin imagery, an unexpected and above chance preponderance of celestial objects (e.g. planets, galaxies, rainbows) was observed in the NN. Although a post-hoc observation, this deserves further investigation. One possibility is that this finding is related to representation of expansive space, consistent with recent findings for neighbouring brain regions^31^.

### The neural representation of imagined objects reflects individual variability in imagery ability and style

Although several semantic and emotional features represented during perception of knives were also robustly represented during knife imagery, this was not the case in the experiment using the dolphin referent. Could it be that different features were being consistently represented during dolphin imagery, but which did not correspond to the features that were tested and were important during perception? Cross-participant consistency in how the referent was represented can be quantified in a feature-agnostic manner, by taking the vector of pattern similarities between the referent and the other items, and correlating these vectors for each pair of participants. The mean cross-participant correlation was compared to a null distribution generated by randomly shuffling the stimulus labels, independently for each participant, repeated 1000 times. While this analysis confirmed significant cross-participant consistency for the perceived and imagined knives and for the perceived dolphin (all p < 0.001), there remained no significant consistency in how the imagined dolphin was related to the other images (p = 0.27). Therefore it seems unlikely that dolphin imagery was evoking a consistent representation that was simply not spanned by those features chosen *a priori*.

Another potential reason for observing relatively low feature tuning of the neural response during dolphin imagery could be that different people imagined the dolphin in different ways, a finding that could have important implications for the use of mental imagery in clinical applications. Two hypotheses were considered. First, some people may have found it more difficult to imagine the dolphin than the kitchen knives (for example because a dolphin is a less familiar object). There was no overall difference in subjective imagery ability between the two groups (scores on the Vividness of Visual Imagery Questionnaire (VVIQ) were matched; t_41_ = −0.92 p > 0.05), but we hypothesised that individuals reporting weak imagery might have struggled to imagine this uncommon object, leading to a correlation between the degree of neural tuning and the ability to generate vivid mental images. For those features exhibiting significant neural tuning during dolphin perception, the difference in tuning between imagery and perception was therefore correlated with VVIQ scores (Fig. 6a). Significant correlations were observed for the features of animacy and image complexity (p < 0.05), along with similar trends for arousal and implied motion (p = 0.07 and p = 0.08 respectively). In all cases, relatively stronger tuning during imagery was observed for participants reporting *less vivid* imagery. The direction of this relationship will be discussed later.

Second, we considered the possibility that whereas different participants tended to imagine the knife image in a similar way (i.e. focusing on the same features), participants might have differed in which features they represented when imagining a dolphin. For example, a bimodal distribution of tuning to “implied motion” might suggest that some participants perceived the dolphin image as static (like a photographic freeze-frame) and others as dynamic (as if they were present in the scene). To investigate this, the distribution across participants of the actual NN feature values was investigated (Fig. 6b). Hartigan’s dip test^43^ was used to assess whether the observed distribution was unimodal, by comparison with a null distribution of the test statistic derived from 10000 bootstrapped samples of similar size, from a Gaussian distribution with similar mean and standard deviation. Again, the five features that showed significant expression in the NN of the perceived dolphin were considered, and tested for unimodality of expression in the NN of the imagined dolphin. Image complexity, emotional arousal, and semantic associations with a dolphin all departed significantly from a unimodal distribution across participants (all p < 0.05). This indicates that the participant sample contained multiple groups of individuals that differed in their style of imagery, specifically by selecting differently along each of these feature dimensions while imagining the dolphin. For comparison, Fig. 6c shows the selected feature distributions during the perception condition, which have means significantly greater than zero, but none of which depart from a unimodal distribution (all p > 0.1).

## Discussion

The current data confirm occipitotemporal involvement in both perception and imagery, with activation pattern similarities encoding image content. Consistent with previous studies, this suggests a meaningful relation between the *form* of neural representations during imagery and perception, while agnostic to what this form might be^24^,^25^,^26^,^27^. By using item-based analyses, in combination with Dynamically Adaptive Imaging^29^, we could assess which object features contributed to this common neural representational form as measured in the multivoxel response pattern. Critically this allows conclusions to be drawn regarding the *content* of the representation, e.g. those stimulus attributes represented similarly in both imagery and perception. Some properties (e.g., semantic associations) showed stronger correspondence than others (e.g., real-world object size) between perception and imagery, and feature tuning was influenced both by the imagined object itself, and by individual differences in style and vividness of imagery.

Perception and imagery dissociate clinically^13^,^34^,^35^,^36^, and theoretical and experimental arguments also highlight differences between them^33^. We find that occipitotemporal representations reflect some of these differences. When items were perceived, sensory, semantic and emotional properties were all represented. However, during knife imagery, much semantic and emotional content was robustly preserved while more sensory details were lost. This is consistent with imagery being supported by abstracted semantic information in long-term memory^44^, drawn from repeated object encounters, but generalizing over episodic details.

Some researchers^26^,^45^ note that prediction of imagined (or verbally evoked) representations, from activity patterns measured during perception, is weaker than prediction in the reverse direction. Cichy and colleagues^26^ hypothesise that this asymmetry may be because imagined representations comprise a subset of the features represented during perception, so that features learned under imagery are likely to be discriminative for representations during perception but not vice versa. Results of experiment 1 are consistent with this prediction (with knife imagery showing significant feature tuning for a subset of the features that were significant during perception), while experiment 2 provides evidence for an alternative, but not mutually exclusive, possibility that encoded features during imagery are more variable (in this case between participants, but potentially also across items).

Concepts can be grouped by common co-occurrence (“thematically”; e.g. “knife”-“sushi”), by type (“taxonomically”, e.g. “knife”-“spoon”), or by other information (for example by similar physical attributes). The left anterior temporal lobe and temporoparietal junction may be specialised respectively for processing taxonomic and thematic information^46^. The current data suggest that occipitotemporal cortex reflects all of these organizational principles, both during perception (knife and dolphin referents), and imagery (knife referent), with tuning observed to thematic properties (e.g. semantic relatedness), taxonomic properties (e.g. animacy) and properties varying across items within a theme or taxon (e.g. emotionality).

Occipitotemporal sensitivity to implied motion in the perceptual condition of both experiments supports previous results^47^,^48^,^49^, which we extend by finding sensitivity to implied motion during knife imagery (negative tuning, i.e. selection of other static objects). Sensitivity to emotional state has also previously been observed in ventral visual regions^9^,^30^,^50^,^51^. The preserved representation of emotional properties during knife imagery, and the bimodal distribution of subjects representing emotional arousal during dolphin imagery, are both consistent with the view that mental imagery is importantly related to emotional experience and its disorders in susceptible individuals^20^, and further imply that occipitotemporal cortex contributes to this link.

Only two semantic properties showed no evidence of preservation during imagery: real-world size and the number of objects in the scene. Recent work suggests that people associate a canonical size to visual objects, represented along the ventral stream^42^,^52^. We replicate this finding during perception, but despite generally preserved tuning to semantic features during knife imagery, there was a significant reduction in coding of real-world size. Size may be represented perceptually to aid computations of distance, and potential physical interactions; during imagery, actual interaction is impossible, and location can be unspecified or pre-specified, compatible with the observed reduction in coding of real-world size. The reduced coding of object numerosity during knife imagery did not reach significance, but is consistent with the observation that while occipitotemporal BOLD signal increases with the number of attended objects, it shows a capacity limit during short-term memory^53^. It is also consistent with the suggestion that serial processing may limit imagery of multiple items, such that the 4 s imagery epochs in the present design may have been too short to generate representations of multiple items within a mental image^54^.

An important difference between perception and imagery is that perception triggers interpretation and identification, whereas people can decide in advance what they will imagine (although hallucinations and flashbacks are potential exceptions). The referent-by-condition interaction in tuning to animacy might reflect this. The observed selection for animate and inanimate items during dolphin and knife perception, respectively, is expected^38^, but during imagery inanimate items were selected in both cases. A similar interaction between category (faces/objects) and condition (imagery/perception) has been observed using EEG^55^. One explanation for this interaction is that activation patterns not only reflect represented features (e.g. animacy), as often implicitly assumed, but mental *processes* being engaged (e.g. identification and interpretation). Identifying other animals is evolutionarily critical, but more challenging than identifying other objects, because animals have relatively few distinguishing features^56^. Therefore, processes such as identification may be recruited more strongly during the perception of living things. This would be compatible with positive and negative tuning during dolphin and knife perception respectively, but negative tuning during imagery of either referent, since identification and interpretation processes becomes irrelevant regardless of category if an imagined referent is known in advance.

Occipitotemporal activation patterns represent different features as different objects are perceived^29^. We suggest that which features a brain region explicitly represents may similarly vary with the object being imagined. This is indicated by a significant interaction between feature set and imagined object, and possibly informs debate on whether imagery maintains depictive^28^ or propositional information^33^, in that the type of information most prominently expressed in a particular mental image might depend on the object being imagined and the goal of the imagery.

Subjective vividness of imagery has long been known to vary substantially between individuals^57^. Nonetheless, there is remarkable concordance between voxel-wise BOLD-signal across individuals imagining verbally-cued items^45^. In retinotopic cortex, VVIQ scores^58^ predict individual differences in activation during imagery^59^, and response pattern similarity between perception and imagery^27^. Here we find that vividness of imagery predicts sensitivity of occipitotemporal cortex to particular stimulus features during imagery. Unexpectedly, relatively *weaker* tuning during imagery was consistently observed for participants who reported *more vivid* imagery. If occipitotemporal representations reflect reactivation of abstracted semantic associations, people reporting weak imagery might rely more on these than people who can vividly imagine specific exemplars and idiosyncratic details. Thus the representations of vivid imagers may rely more on earlier visual cortex than on the current occipitotemporal region of interest. Representations of people reporting vivid imagery may also be more variable across individuals if they focus on different details, or elaborate their images based on different past experiences. This is consistent with the view that effective imagery can be thought of not as a “faithful replica of the physical object” but a “refined abstraction” whose “incompleteness…[is] a positive quality, which distinguishes the mental grasp of an object from the physical nature of that object itself”^60^. Finally, during dolphin imagery, tuning to several features (that were consistently coded during perception) was found to be distributed bimodally across participants, demonstrating that distinct participant groups differ in their imagery style, and extending the recent finding that response patterns in occipitotemporal cortex predict differences between individuals’ perceptual judgements^61^.

Adaptive similarity search^29^ efficiently samples a large stimulus set to answer questions focused on local neighbourhoods, important if neural representations are inhomogeneous across stimulus space, as observed previously^29^. The current study investigated in great detail the feature content of occipitotemporal cortex during perception and imagery, but only for two objects. Future work might extend this to a greater number of referents, or use our findings to guide a broader but shallower investigation. Another trade-off in the current approach is optimisation for a given ROI. Occipitotemporal cortex was chosen because of its reliable involvement in both imagery and perception^24^,^25^,^26^,^27^,^62^ and its coding of multiple visual attributes^29^. However, the occipitotemporal ROI is only a subset of cortex implicated in perception and imagery^22^, and other features may be represented elsewhere in the brain. Imagery additionally activated hippocampus, parieto-occipital cortex and the insula, and sometimes engages retinotopic areas^28^. Some of these regions, such as the hippocampus, may represent content-specific properties^63^, while others may serve task-general roles^64^. The current approach offers a tool to measure and contrast tuning to different object properties across these regions. It remains unclear whether feature tuning is computed within the region of interest, or reflects upstream input. For example, frontal cortex may process emotional imagery and subsequently modulate occipitotemporal representations^9^,^30^. Methods with finer temporal resolution may help elucidate this^48^.

Although our methods allow for the examination of many image features, multiple-comparison correction limits statistical power. Occipitotemporal cortex likely represents further properties of perceived and imagined images, not present amongst the stimuli or untested here, such as visual field location^26^. Caution is also warranted in inferring from above-chance expression in the NN, of features defined *a priori*, that these are actually what is being represented. For example, although the present three-level scale for “animacy” revealed significant tuning in three of four conditions, it is likely that occipitotemporal cortex represents finer gradations along a dimension from less to more human-like^65^. Similarly, brain responses may be better described by other, possibly unintuitive, object features or computation processes that covary with the features chosen for investigation.

Decoding mental states using neuroimaging attracts much interest, and can have profound clinical impact^21^. Experiments typically identify which of a few pre-specified stimuli or tasks are being represented. To infer internal states more generally, a recent approach has been to train classifiers on neural response patterns to known stimuli, and attempt to predict responses to novel stimuli, or to predict a novel stimulus from its response^66^,^67^. Adaptive search through stimulus space provides a complementary strategy, perhaps especially powerful in combination with parameterised spaces, as demonstrated in monkey electrophysiology^68^. However, the current results suggest caution in interpreting whether or not it is possible to decode a mental image. Firstly, since the relationship between perceived and imagined representations depended on the particular object, different mental images may vary in their decodability, unless these dependencies are characterised. Secondly, since neural tuning covaried with VVIQ scores, and exhibited across-participant clustering, imagery is unlikely to be equally decodable across people, and individual differences in style and ability should be considered.

In conclusion, the current results demonstrate that it is possible to measure the content of a mental image by analysing patterns of brain activity. Specifically, we were able to characterize imagined semantic and emotional properties represented in occipitotemporal cortex. Despite similar response patterns during perception and imagery, this correspondence is shown to differ across features, with only a subset of perceptually represented properties being consistently represented during imagery. Importantly, this feature tuning depends upon both the imagined object, and individual differences in personal style and vividness of imagery.

Roger Shepard commented that mental images “have often appeared to play a crucial role in processes of scientific discovery, [but] have themselves seemed to remain largely inaccessible to those very processes”^4^. By characterising mental image content via its representation in occipitotemporal cortex, we offer a new approach to shed light on this internal world, and increase its accessibility to scientific discovery.

## Methods

### Stimuli and task

To generate the stimulus set, two images were selected from each of 100 categories in the Caltech256 database^69^. In each experiment, one image from the stimulus set was selected as a “referent image”. An image of kitchen knives was selected as the referent stimulus in experiment 1, and an image of a dolphin in experiment 2. The referent images were chosen to be perceptually, semantically, and emotionally distinct from each other. All stimuli were displayed sequentially in the centre of a mid-grey screen, and resampled to occupy a constant visual area (equivalent to a square of side 6° of visual angle). Presentation used DirectX and VB.net 2008 Express Edition, running on a Windows PC. Stimuli were back-projected onto a screen and viewed through a mirror.

Prior to entering the scanner, participants completed the Vividness of Visual Imagery Questionnaire VVIQ^58^,^59^, to provide an individual measure of subjective imagery ability, and to allow participants to practise generating vivid mental images. Participants were next presented with a referent image (a set of knives in experiment 1, and a dolphin in experiment 2) and were given as long as they wanted to memorise it and to practise imagining it as vividly as possible. Participants then completed two half-hour scanning sessions: An imagery session in which they imagined the previously memorized referent image whenever visually cued with an empty rectangle, and a perceptual session in which instead of the empty rectangle the referent image was displayed on the screen. In both cases, these referent events were interspersed amongst a stream of other stimuli, to which participants were simply required to pay attention whilst maintaining central fixation. The order of the two sessions was counterbalanced across participants.

### Participants

Each experiment tested a different group of 22 participants, who all reported normal or corrected-to normal vision, and no history of psychological or neurological impairment. In experiment 1 (knife referent), one block was excluded because the participant fell asleep; participants (11 female) were aged between 18 and 32 (mean 23). In experiment 2 (dolphin referent), one participant was excluded due to frequent movement of >3 mm; the remaining participants (13 female) were aged between 18 and 38 (mean 25). A separate group of thirteen participants provided subjective ratings of semantic and emotional properties of all images, using a five-point scale (Table 1). All participants gave informed, written consent, and were paid for taking part. The study was approved by the Cambridge Psychology Research Ethics Committee and carried out in accordance with the approved guidelines.

### MRI acquisition

Scanning was performed using a Siemens 3T Tim Trio at the MRC CBU in Cambridge, UK. Functional magnetic resonance imaging (fMRI) acquisitions used Echo-Planar Imaging (TR  =  1 s, TE  =  30 ms, FA  =  78 degrees) with a matrix size of 64 × 64 and in-plane voxel size of 3 × 3 mm. There were 16 slices with a rectangular profile, 3 mm thick and separated by 0.75 mm. Slices were oriented to cover the inferior surface of the occipital and temporal lobes. Each acquisition began with 18 dummy scans during which a countdown was shown to the participant. An MPRAGE sequence (TR  =  2.25 s, TE  =  2.98 ms, FA  =  9 degrees) was used to acquire an anatomical image of matrix size 240 × 256 × 160 with a voxel size of 1 mm^3^. Structural and functional data analyses were performed in real-time using custom scripts written in Matlab (2006b; The MathWorks Inc.). SPM5 (Wellcome Department of Imaging Neuroscience, London, UK) was used to perform image pre-processing and statistical modelling. The analysis was run on a stand-alone RedHat Enterprise 4 Linux workstation. As each image volume was acquired, it was motion-corrected and the time-course was high-pass filtered (cut-off 128 s). No spatial smoothing or slice-time adjustment were applied.

### Online adaptive stimulus selection

**Figure 1:**
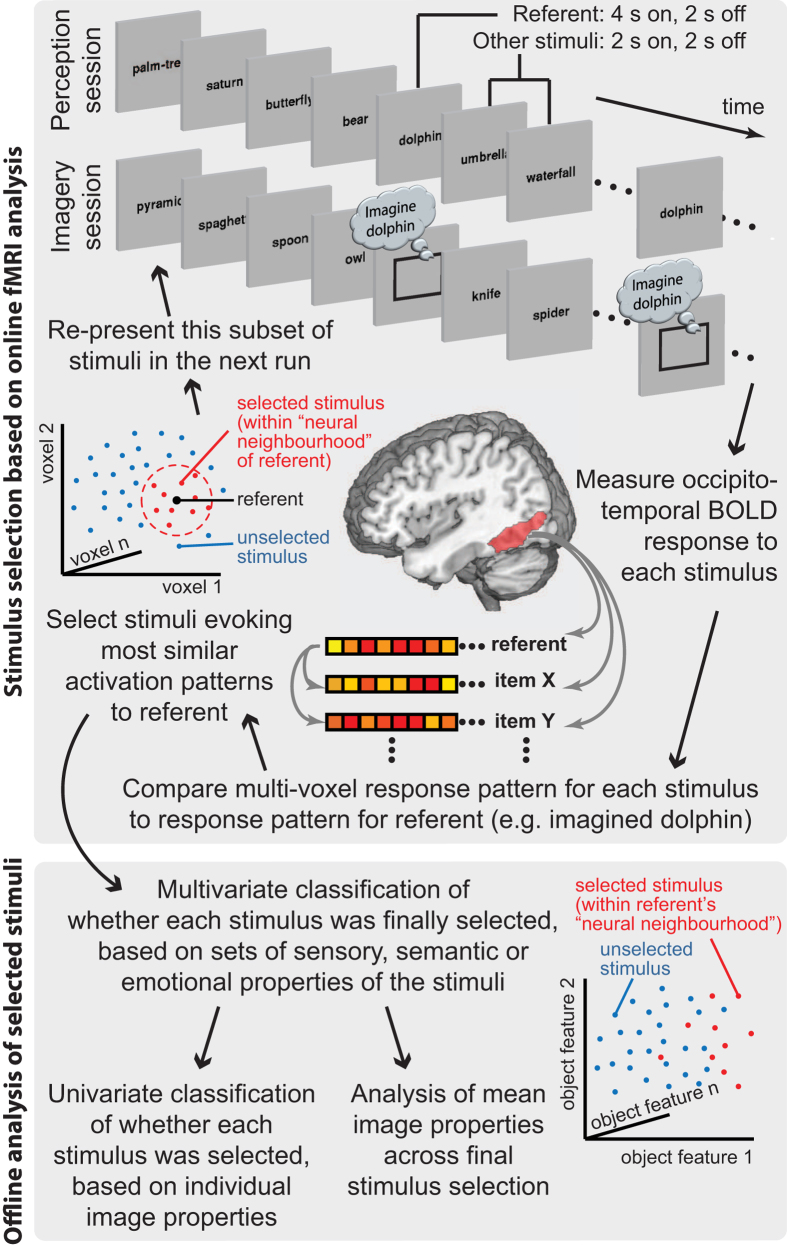
Overview of experimental procedure. (Top) Example sequence of events in the two conditions, and stimulus selection procedure based on online fMRI analysis. Actual stimuli were coloured images, represented here by their category label. (Bottom) Offline analyses of selected stimulus sets.

**Figure 2:**
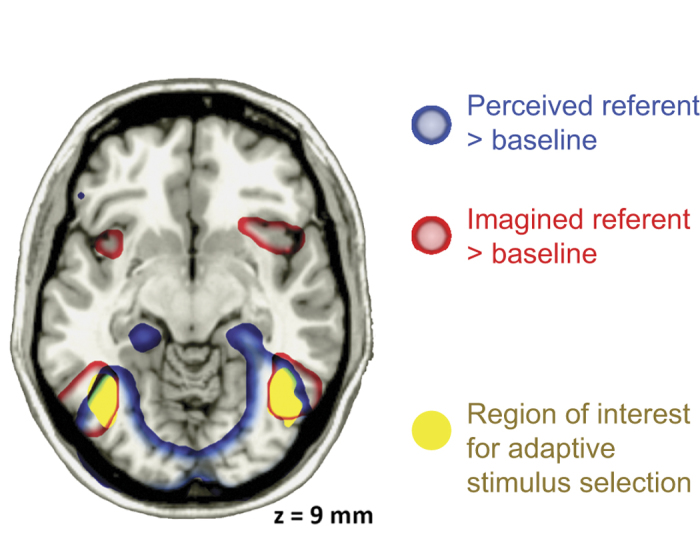
Univariate BOLD response to referent items that are perceived (blue contour) or imagined (red contour). Activations are significant at a false discovery rate of 0.05. The region of interest, defined a priori, is superimposed in yellow.

Dynamically Adaptive Imaging^29^, a recently developed fMRI methodology, was used to iteratively select from the 200 stimuli those that evoked the most similar pattern of brain activity to the referent stimulus (Fig. 1, top). All 200 images were presented in the initial run, once each, in random order. Images appeared for 2 s each, separated by a 2 s blank interval. Every 4th image was followed alternately by either a blank screen or the referent stimulus, lasting 4 s, and again separated by a 2 s gap. In the imagery session the referent stimulus was simply a black rectangle cueing the participant to imagine the relevant image as vividly as possible. An occipitotemporal region of interest (ROI) was predefined from an independent study^29^ that used a functional localizer to identify object-selective cortex^70^. This region is known to encode both semantic information and lower-level sensory features^29^, and is activated in an object-specific manner during both perception and imagery^24^,^25^,^62^. The ROI was inverse-normalised from the space of the MNI template back to the native space of each participant’s brain (with the resultant number of voxels ranging from 223–371). At the end of each run, data extracted from this ROI were submitted to a standard general linear model. 201 regressors modelled each stimulus and the referent, formed from boxcars during each presentation of that item (across all preceding runs), convolved with the canonical hemodynamic response as defined by SPM. Additional regressors were used to model and remove residual effects of participant motion, and a temporal high-pass filter was applied to the data and model (128 s cut-off). Using multivoxel pattern analysis (MVPA), a subset of stimuli was selected, whose evoked activity patterns were most similar to that of the referent. The similarity metric was the Spearman correlation across voxels between the patterns of betas from the fitted model. Following completion of each run there was a 24 s interval, and the selected images were then presented again in a new run. This process was iterated to converge on the ten most similarly represented images, which defined the referent’s “neural neighbourhood” (NN). In each session there were six runs, with the number of (non-referent) stimuli in each being 200, 31, 25, 20, 16 and 13, (presented once each), as optimized by simulations prior to the experiments^29^. In this way, neuroimaging was primarily used online to generate a neural neighbourhood of stimuli, and not for subsequent analyses.

### Offline analysis of feature-tuning

Each referent was characterized by its NN, the set of ten items that evoked the most similar patterns of activity to it. To test whether given features or sets of features contributed to an item having a similar neural representation to the referent, a classifier was trained to predict, on the basis of an item’s features, whether or not it would make it into the NN. The features tested included simple, space-invariant visual properties computed directly from the images, as well as subjectively-rated semantic and emotional characteristics, as listed in Table 2. A quadratic-discriminant naïve Bayes classifier was chosen (implemented by the “diagQuadratic” option of the “classify” function in MATLAB), because it allows for covariance to differ between categories, as might be expected for our asymmetric within-NN vs. outside-NN discrimination. A leave-one-out strategy was used to assess generalization across items. To maximise the amount of data contributing to training, on each fold the classifier was tested on a single item from a single participant, after training on all other items over all participants; this was then repeated for each item and each participant. To summarise the overall performance of the classifier, signal detection theory’s d’ was used. (The more common measure of percent correct classification would be misleading with our asymmetric categories, because a classifier that never selected “within-NN” could achieve 95% accuracy despite having a sensitivity of zero.) Significance was assessed using a permutation test, where the null distribution of the d’ statistic was computed from 1000 iterations in which the items’ category labels (within-NN vs. outside-NN) were randomly reassigned. Actual d’ was compared to the 95^th^ percentile of this null distribution.

To decompose the pattern of selectivity into tuning along individual feature dimensions, whilst controlling the number of statistical tests, a hierarchical approach was taken. First, features were broken down into three broad sets: “sensory” (computed objectively from the image pixels or from the contour of the focal objects), “semantic” (subjectively rated properties of the image) or “emotional” (subjectively rated emotions evoked by the images). Separate, multivariate classifiers were trained and tested using just the features in a given set. For feature sets where significant prediction was found, separate univariate classifiers were then trained using each feature within this set individually. The Holm-Bonferroni procedure was used to control the false positive rate across multiple comparisons within each branch.

The classifier analysis revealed which features significantly predicted NN membership (e.g., emotional valence), but not whether positive or negative values of each feature were being selected for. To investigate this more directly, the direction of tuning of the NN for each feature was assessed by testing whether that feature was found in the NN more or less than would be expected by chance. For each feature, the distribution of values was standardized to a z-score across all 200 images. This allowed us to test how significant the expression of a feature in the NN was, by measuring the mean value of this feature across the ten images in the NN, and comparing this to zero (which would be expected if images were being selected at random).

### Testing pattern consistency across repeated stimulus presentations

Because the analysis depends on the reliability of fMRI patterns for each stimulus, a standard MVPA classification was used to decode the identity of stimuli that were repeated in four or more runs. For each participant, a linear support vector machine was used to classify each pair of stimuli, training and testing on separate runs. Classification accuracy was averaged across cross-validation splits and stimulus pairs, and compared to chance using t-tests across participants.

### Multi-dimensional scaling

**Figure 3:**
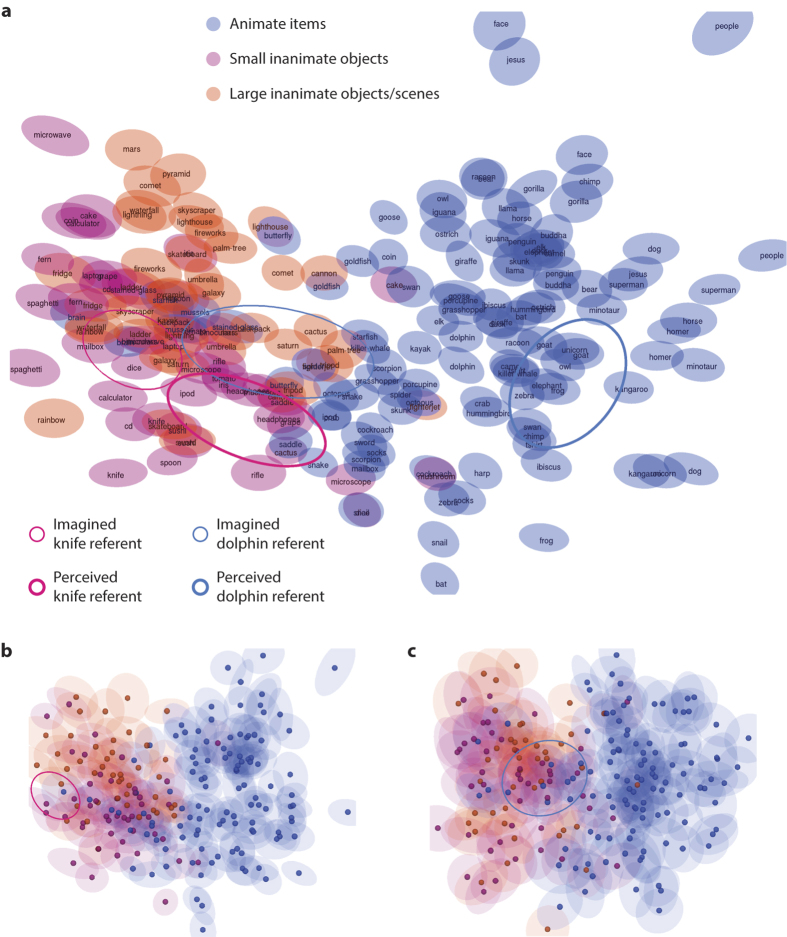
The representational space of the 200 stimuli based on their occipitotemporal activation patterns at the end of the first run. The first two dimensions of this space are illustrated. The DISTATIS extension of multidimensional scaling was used to optimally combine participants; variability is indicated by 95% confidence ellipses, coloured according to a tripartite classification into animate items, and small or large inanimate objects. Panel (**a**) combines data across the four conditions, and the image categories from the Caltech 256 dataset are superimposed on the ellipses. The lower panels show data for the two imagery conditions: experiment 1, knife imagery (**b**), and experiment 2, dolphin imagery (**c**). Elliptical outlines are bootstrapped 95% confidence ellipses illustrating the location of each referent’s neural neighbourhood, defined on the first two dimensions and averaged across subjects.

**Figure 4:**
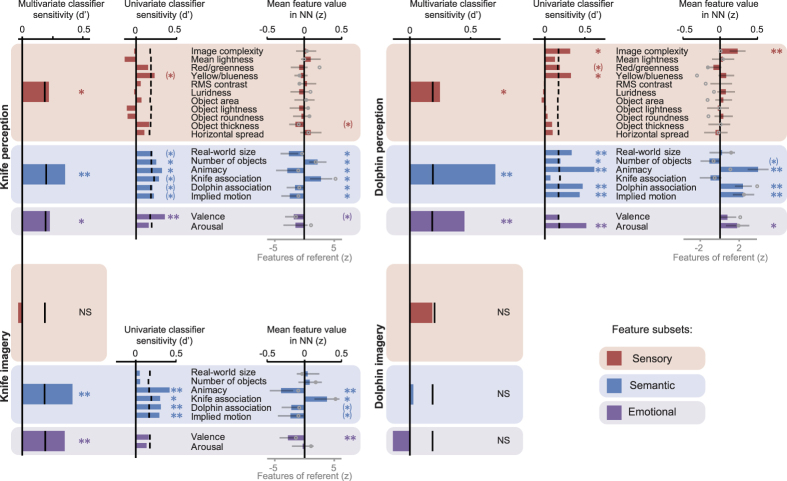
Information represented within the neural neighbourhood of perceived and imagined images. Expression of each feature within the neural neighbourhoods of experiment 1 (left panels) and experiment 2 (right panels). Upper panels are for the perception conditions and lower panels for the imagery conditions. **p < 0.01 with Holm-Bonferroni correction for number of tests in category; *p < 0.05 Holm-Bonferroni; *p < 0.05 uncorrected. For the classifier analysis, black lines indicate the 95^th^ percentile of the null distribution. For the plots of mean feature values in the NN, error bars represent 95% confidence intervals across participants, and the grey circles show the feature values for each referent.

**Figure 5:**
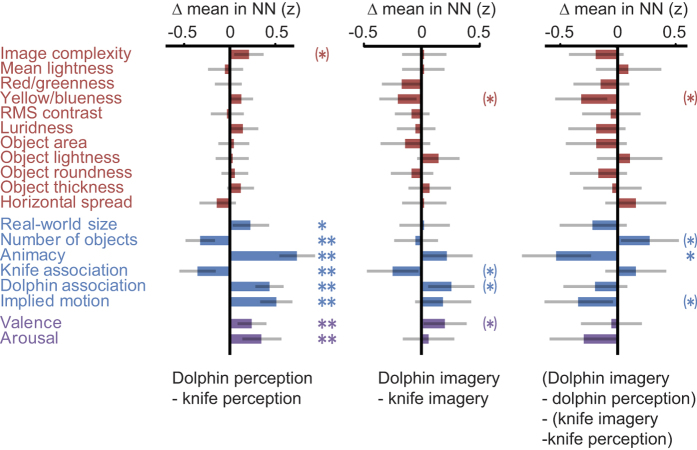
Object-specific feature coding during perception (left), imagery (middle) and their interaction (right). **p < 0.01 with Holm-Bonferroni correction for number of tests in category; *p < 0.05 Holm-Bonferroni; *p < 0.05 uncorrected. Error bars represent 95% confidence intervals across participants.

**Figure 6:**
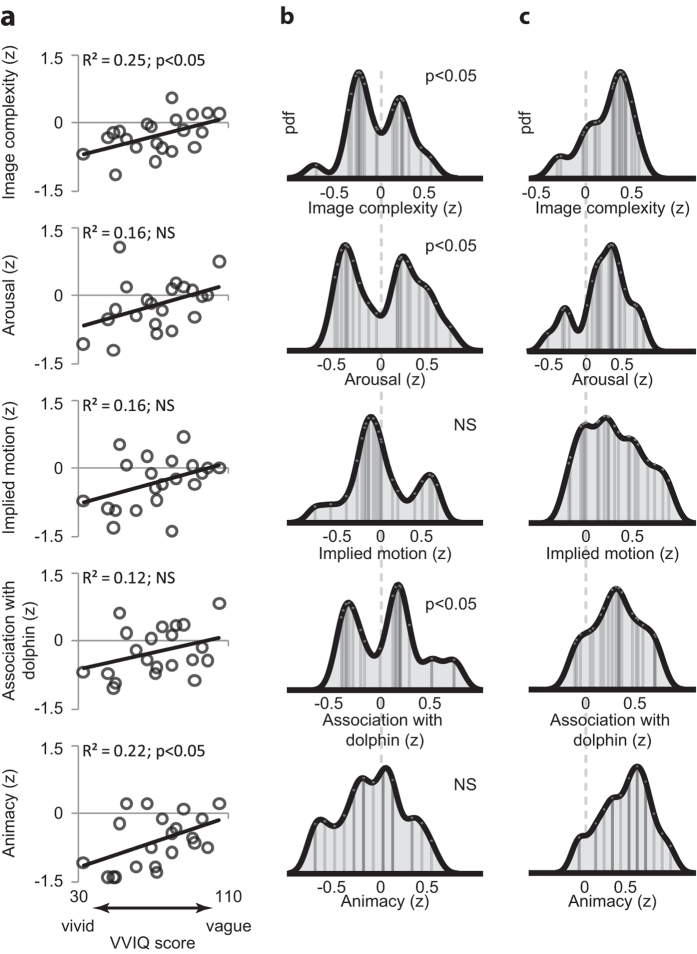
Individual differences in neural feature tuning during imagery. (**a**) Correlation of subjective imagery vividness with neural feature tuning, during dolphin imagery relative to perception. (**b**) Kernel density estimates (bandwidth = 0.1), of the distribution across participants, of mean feature values within the NN, during dolphin imagery. Significance indicates Hartigan’s dip test for departure from unimodality. (**b**) Corresponding kernel density estimates during dolphin perception.

To illustrate the representational space of the 200 stimuli (Fig. 3), multi-dimensional scaling (MDS) was used to convert the voxel pattern correlation distances between all pairs of stimuli into points on a 2D plane, whose euclidean distances approximate the distances in the original multidimensional space. DISTATIS was used to optimally combine data across subjects and conditions^40^, with variability indicated by 95% confidence ellipses. The special status of the referents means that absolute distances between the referents and the 200 comparison stimuli are not directly comparable to distances amongst the 200 stimuli themselves. The location of each referent in the representational space is therefore illustrated as the mean coordinate of the items in its mean neural neighbourhood, defined on the first two dimensions, with bootstrapped 95% confidence ellipses illustrating variability across subjects. Procrustes transformation was used to rigidly align representational spaces across the conditions and experiments.

## Additional Information

**How to cite this article**: Mitchell, D. J. and Cusack, R. Semantic and emotional content of imagined representations in human occipitotemporal cortex. *Sci. Rep*. **6**, 20232; doi: 10.1038/srep20232 (2016).

## Figures and Tables

**Table 1:** Neural neighbourhoods of images evoking the most similar patterns, on average, to each perceived or imagined referent.

***Knife Perception***		***Knife Imagery***		**Dolphin Perception**		**Dolphin imagery**	
**Bat 007.0022**	(0.317)	*Knife 125.0034*	(0.154)	**Unicorn 236.0032**	(0.405)	Snail 189.0002	(0.178)
*Knife 125.0034*	(0.291)	*Sword 209.0017*	(0.146)	**Kangaroo 121.0051**	(0.376)	Rainbow 170.0060	(0.171)
*Mushroom 147.0045*	(0.277)	*Knife 125.0006*	(0.138)	**Dog 056.0028**	(0.373)	**Killer-whale 124.0001**	(0.167)
*Sushi 206.0072*	(0.274)	*Microscope 141.0021*	(0.125)	**Superman 208.0008**	(0.365)	Saturn 177.0029	(0.160)
*Spoon 199.0040*	(0.273)	Pyramid 167.0002	(0.124)	**Camel 028.0082**	(0.354)	*Brain 020.0058*	(0.158)
**Zebra 250.0083**	(0.265)	*Mushroom 147.0002*	(0.123)	**Bat 007.0022**	(0.332)	Galaxy 082.0050	(0.152)
*Sushi 206.0058*	(0.260)	**Butterfly 024.0098**	(0.123)	**Homer 104.0003**	(0.330)	*Umbrella 235.0042*	(0.139)
**Snake 190.0047**	(0.258)	**Ostrich 151.0017**	(0.119)	**Zebra 250.0083**	(0.328)	*Laptop 127.0002*	(0.138)
**Llama 134.0001**	(0.253)	*Umbrella 235.0042*	(0.114)	**Swan 207.0007**	(0.323)	Tripod 231.0039	(0.136)
*Socks 194.0083*	(0.252)	**Snail 189.0052**	(0.112)	**Horse 105.0073**	(0.318)	**Elephant 064.0039**	(0.136)

Category names are followed by the image ID in the Caltech 256 dataset. Font style indicates tripartite classification into animals (bold), small inanimate objects (*italic*), and large inanimate objects (regular). Mean pattern correlation with the referent is given in parentheses.

**Table 2:** Derivation of feature scores.

**Feature**		**Derivation**	**Lowest scoring items**	**Highest scoring items**
Sensory	Image complexity	Jpeg file size^71^	tripod_231_0039	elk_065_0039
			mars_137_0003	fern_068_0023
			llama_134_0019	fern_068_0002
	Mean lightness	Mean across image of L* channel in L*a*b* colour space	saturn_177_0082	ipod_117_0042
			comet_044_0032	unicorn_236_0032
			saturn_177_0029	tripod_231_0039
	Red/green balance	Mean across image of a* channel in L*a*b* colour space	hummingbird_113_0008	homer_104_0003
			grape_092_0152	microscope_141_0006
			kangaroo_121_0057	tomato_221_0007
	Yellow/blue balance	Mean across image of b* channel in L*a*b* colour space	killer-whale_124_0001	sword_209_0017
			dolphin_057_0009	tomato_221_0007
			killer-whale_124_0066	homer_104_0003
	RMS contrast	Standard deviation across image for L* channel	comet_044_0032	laptop_127_0002
			frog_080_0065	headphones_101_0056
			fighterjet_069_0044	backpack_003_0036
	Luridness	Mean standard deviation across image for a* and b* channels	elk_065_0093	homer_104_0019
			tripod_231_0039	microscope_141_0006
			dog_056_0028	homer_104_0003
	Area of focal object(s)	Number of pixels occupied by focal object(s)†	comet_044_0022	coin_043_0008
			kayak_122_0009	racoon_168_0013
			lightning_133_0135	tomato_221_0007
	Object lightness	L* difference between focal object(s) and background†	coin_043_0026	laptop_127_0043
			headphones_101_0056	cake_026_0008
			socks_194_0083	mars_137_0108
	Object roundness	Ratio of eigenvalues of pixel coordinates of focal object(s)†	comet_044_0032	spaghetti_196_0019
			spoon_199_0002	coin_043_0026
			knife_125_0034	mars_137_0108
	Object thickness	Area of focal object(s)† scaled by square of its perimeter	fern_068_0023	mars_137_0108
			lightning_133_0013	spaghetti_196_0019
			fireworks_073_0016	coin_043_0026
	Horizontal spread	Orientation* (1-roundness) (-π/4 = vert., π/4 = horiz.)†	sword_209_0040	pyramid_167_0001
			harp_098_0058	lightning_133_0013
			superman_205_0014	skateboard_185_0002
Semantic	Real-world size	Ranked size, estimated by researcher	dice_055_0062	saturn_177_0082
			coin_043_0008	galaxy_082_0029
			coin_043_0026	galaxy_082_0050
	Number of focal objects	Rated by researcher (0 = one, 1 = two, 2 = more than two)	backpack_003_0036	sushi_206_0072
			backpack_003_0145	tomato_221_0007
			bat_007_0069	zebra_250_0048
	Animacy	0 = inanimate, 1 = ambiguous, 2 = animate	backpack_003_0036	swan_207_0025
			backpack_003_0145	zebra_250_0048
			binoculars_012_0001	zebra_250_0083
	Semantic association with knives	Rated by participants on 5-point scale (5 = highly related)	bat_007_0069	sword_209_0040
			bear_009_0021	knife_125_0034
			bear_009_0071	knife_125_0006
	Semantic association with dolphin	Rated by participants on 5-point scale (5 = highly related)	backpack_003_0036	killer-whale_124_0066
			backpack_003_0145	dolphin_057_0034
			cake_026_0008	dolphin_057_0009
	Implied motion	Rated by participants on 5-point scale (5 = most dynamic)	backpack_003_0036	fighterjet_069_0003
			backpack_003_0145	fighterjet_069_0044
			binoculars_012_0001	killer-whale_124_0001
Emotional	Subjective valence	Rated by participants on 5-point scale (5 = most pleasant)	spider_198_0001	killer-whale_124_0001
			cockroach_040_0022	rainbow_170_0060
			cockroach_040_0122	dolphin_057_0009
	Subjective arousal	Rated by participants on 5-point scale (5 = most arousing)	fridge_171_0001	spider_198_0033
			microwave_142_0069	fighterjet_069_0044
			microwave_142_0081	scorpion_179_0071

The three highest and lowest scoring items on each feature are listed by category name followed by the image ID in the Caltech 256 dataset.

^†^Contour of focal object(s) was traced by experimenter.
